# Tezepelumab in real-world US patients with severe asthma across phenotypes and underrepresented populations: the phase 4 PASSAGE study

**DOI:** 10.1093/ajrccm/aamag225

**Published:** 2026-05-18

**Authors:** Njira L Lugogo, Praveen Akuthota, Kaharu Sumino, Autumn F Burnette, Sameer K Mathur, Andrew W Lindsley, Jean-Pierre Llanos, Claudio Marchese, Christopher S Ambrose, Benjamin Emmanuel

**Affiliations:** Division of Pulmonary and Critical Care Medicine, Department of Medicine, University of Michigan, Ann Arbor, MI, United States; Division of Pulmonary, Critical Care and Sleep Medicine, University of California, San Diego, La Jolla, CA, United States; Division of Pulmonary and Critical Care Medicine, Department of Medicine, Washington University School of Medicine, St Louis, MO, United States; Division of Allergy and Immunology, Howard University, Washington, DC, United States; Division of Allergy, Pulmonary and Critical Care Medicine, Department of Medicine, University of Wisconsin–Madison, Madison, WI, United States; US Medical Affairs, Amgen, Thousand Oaks, CA, United States; Global Medical Affairs, Amgen, Thousand Oaks, CA, United States; Respiratory and Immunology, BioPharmaceuticals R&D, AstraZeneca, Barcelona, Spain; Respiratory and Immunology, BioPharmaceuticals Medical, AstraZeneca, Gaithersburg, MD, United States; Respiratory and Immunology, BioPharmaceuticals Medical, AstraZeneca, Gaithersburg, MD, United States

**Keywords:** phenotype, real-world, severe asthma, tezepelumab, underrepresented populations

## Abstract

**Rationale:**

Clinical trials of severe asthma therapies often exclude or underrepresent key patient populations.

**Objectives:**

To evaluate the effectiveness and safety of tezepelumab in a diverse, real-world US population with severe, uncontrolled asthma (SUA).

**Methods:**

PASSAGE was a phase 4, multicenter, single-arm, open-label, 12-month study enrolling patients with SUA (≥12 years old), including different phenotypes (blood eosinophil count ≥300 or <300 cells/µL, with or without allergy) and underrepresented populations (Black/African American patients, adolescents, those with SUA with comorbid mild-to-moderate chronic obstructive pulmonary disease, people with a significant smoking history [≥10 pack-years]). The primary outcome was the annualized asthma exacerbation rate (AAER) in the 12 months before (baseline period) and after (treatment period) tezepelumab initiation.

**Measurements and Main Results:**

Among 286 participants, AAER decreased by 70% (95% CI, 63%-75%) from 2.88 (baseline period) to 0.87 (treatment period) and by 54% to 77% across phenotypes and underrepresented populations. At week 52, least-squares mean pre-bronchodilator FEV_1_ increased from baseline by 0.122 L (95% CI, 0.07-0.17) overall and by 0.212 L (95% CI, 0.15-0.28) among participants with percent predicted pre-bronchodilator FEV_1_ ≤80% at baseline. Clinically meaningful improvements in Asthma Control Questionnaire-6, Asthma Impairment and Risk Questionnaire, and St George’s Respiratory Questionnaire scores were observed in 51% to 91% of participants across phenotypes and underrepresented populations at week 52. No new safety signals were identified.

**Conclusions:**

The PASSAGE study of a diverse, real-world US population with SUA treated with tezepelumab demonstrated substantial reductions in asthma exacerbations across phenotypes and underrepresented populations as well as clinically meaningful improvements in lung function, asthma control, and health-related quality of life.

**Clinical trial registration:**

www.clinicaltrials.gov (NCT05329194).

At a Glance Commentary
**Scientific Knowledge on the Subject:** Randomized controlled trials (RCTs) typically apply strict eligibility criteria, which exclude a large proportion of real-world patients. In severe asthma, RCTs often exclude or underrepresent key groups, including varied asthma phenotypes, racially diverse populations, adolescents, and patients with comorbid chronic obstructive pulmonary disease (COPD) or with significant smoking history. Consequently, real-world evidence is needed to complement RCT results. Tezepelumab, an anti-thymic stromal lymphopoietin monoclonal antibody was approved in 2021 for U.S. patients with severe asthma.

**What This Study Adds to the Field:** PASSAGE was a phase 4, single-arm, 12-month study that assessed the effectiveness and safety of tezepelumab in a diverse, real-world cohort of U.S. patients with severe, uncontrolled asthma. The study included protocol-driven enrollment of multiple prespecified asthma phenotypes defined by blood eosinophil count and allergy status, and historically underrepresented or excluded populations, specifically Black/African American patients, adolescents, patients with comorbid mild-to-moderate COPD, and patients with ≥10 pack-years smoking history. Across these asthma phenotypes and underrepresented populations, tezepelumab recipients had substantial reductions in asthma exacerbations. There were also clinically meaningful improvements in lung function, asthma control, and health-related quality of life at 52 weeks post-initiation of tezepelumab. No new safety concerns were identified.

## Introduction

Real-world evidence of the effectiveness and safety of treatments is important to complement randomized controlled trial (RCT) results and establish their generalizability. RCTs enroll participants based on extensive inclusion and exclusion criteria, which greatly limit the proportion of eligible patients. In severe asthma, where there is considerable heterogeneity of inflammatory phenotypes (such as the presence/absence/overlap of eosinophilic and allergic inflammation), symptoms, and comorbidities, up to 90% of real-world patients may be ineligible for enrollment in RCTs.^1^^,^^2^ Additionally, regulatory standards generally exclude patients with ≥10 pack-years of smoking history and patients with comorbid chronic obstructive pulmonary disease (COPD).^3^^,^^4^ RCTs also often fail to recruit meaningful numbers of adolescents and patients from diverse racial and ethnic backgrounds.^5^^,^^6^ For example, Black/African American patients have historically been underrepresented in RCTs of biologic therapies for severe asthma,^7^ despite having a disproportionately high prevalence of, and more difficult to treat, severe asthma^8^ and having approximately 2 times more asthma-related emergency department (ED) visits and deaths^9^ than White patients.

Tezepelumab, a human monoclonal antibody that blocks thymic stromal lymphopoietin,^10^ was approved as an add-on maintenance treatment for severe asthma by the US Food and Drug Administration in 2021 and by the European Medicines Agency in 2022.^11^^,^^12^ In the global phase 2b PATHWAY (NCT02054130) and phase 3 NAVIGATOR (NCT03347279) studies, tezepelumab significantly reduced the annualized asthma exacerbation rate (AAER) versus placebo in patients with severe, uncontrolled asthma (SUA). Lung function, asthma control, and health-related quality of life (HRQoL) also improved with tezepelumab versus placebo.^13^^,^^14^ However, there is limited information available describing outcomes with tezepelumab post-approval and its use in real-world patients with severe asthma.

PASSAGE (NCT05329194) was a phase 4, post-authorization study designed to assess the effectiveness and safety of tezepelumab in a diverse, real-world cohort of US patients with SUA. The study included protocol-driven enrollment of multiple prespecified asthma phenotypes defined by blood eosinophil count (BEC) and allergy status, and underrepresented populations, specifically Black/African American patients, adolescents, SUA patients with comorbid mild-to-moderate COPD, and patients with significant smoking history. Following the publication of an interim analysis in 2025,^15^ here, we report the final results of PASSAGE.

## Methods

### Study design and participants

PASSAGE was a phase 4, post-authorization, multicenter, single-arm, prospective, open-label, 12-month study conducted in a real-world patient population (≥12 years old) in the United States. Participants had a history of severe asthma requiring medium- to high-dose inhaled corticosteroids per Global Initiative for Asthma 2021 guidelines (daily dose of fluticasone propionate >250-500 to >500 µg or equivalent),^16^ with additional controller medications for at least 12 months before enrollment and a documented history of at least 2 asthma exacerbations during the previous year.^17^ Participants with any contraindication to tezepelumab or a comorbid diagnosis of severe or very severe COPD (per Global Initiative for Chronic Obstructive Lung Disease [GOLD] guidelines) were excluded from the study.^18^ Study inclusion and exclusion criteria were designed to recruit a real-world population of specialist-treated patients with SUA (for full inclusion and exclusion details, see the online supplement).

The study was conducted at 32 sites across the United States from April 2022 to September 2024. The study aimed for the protocol-driven enrollment of approximately 50 participants in each of 4 prespecified asthma phenotypes defined by both the highest documented BEC in the previous year and allergy status (sensitization to any perennial aeroallergen): nonallergic asthma with BEC <300 cells/µL, allergic asthma with BEC <300 cells/µL, nonallergic asthma with BEC ≥300 cells/µL, and allergic asthma with BEC ≥300 cells/µL. The 4 underrepresented populations targeted for protocol-driven enrollment of approximately 50 participants per group were individuals who identify as Black/African American, adolescents (12-17 years old), SUA patients with a comorbid diagnosis of mild-to-moderate COPD consistent with GOLD 2021 guidelines^18^ as confirmed by the investigator (specific clinical testing not required), and individuals with a smoking history of ≥10 pack-years (people who currently smoke or formerly smoked). Participants could belong to more than one of these underrepresented populations; classification was not mutually exclusive.

After a screening period of up to 4 weeks, enrolled participants received their first dose of tezepelumab 210 mg subcutaneously at week 0 and thereafter every 4 weeks for 52 weeks. The index date was defined as the date that a participant received their first tezepelumab dose. In total, participants received up to 13 subcutaneous injections, with the last dose administered at week 48. The baseline and treatment periods were the 12 months before and after the index date, respectively (Figure S1).

**Figure 1:**
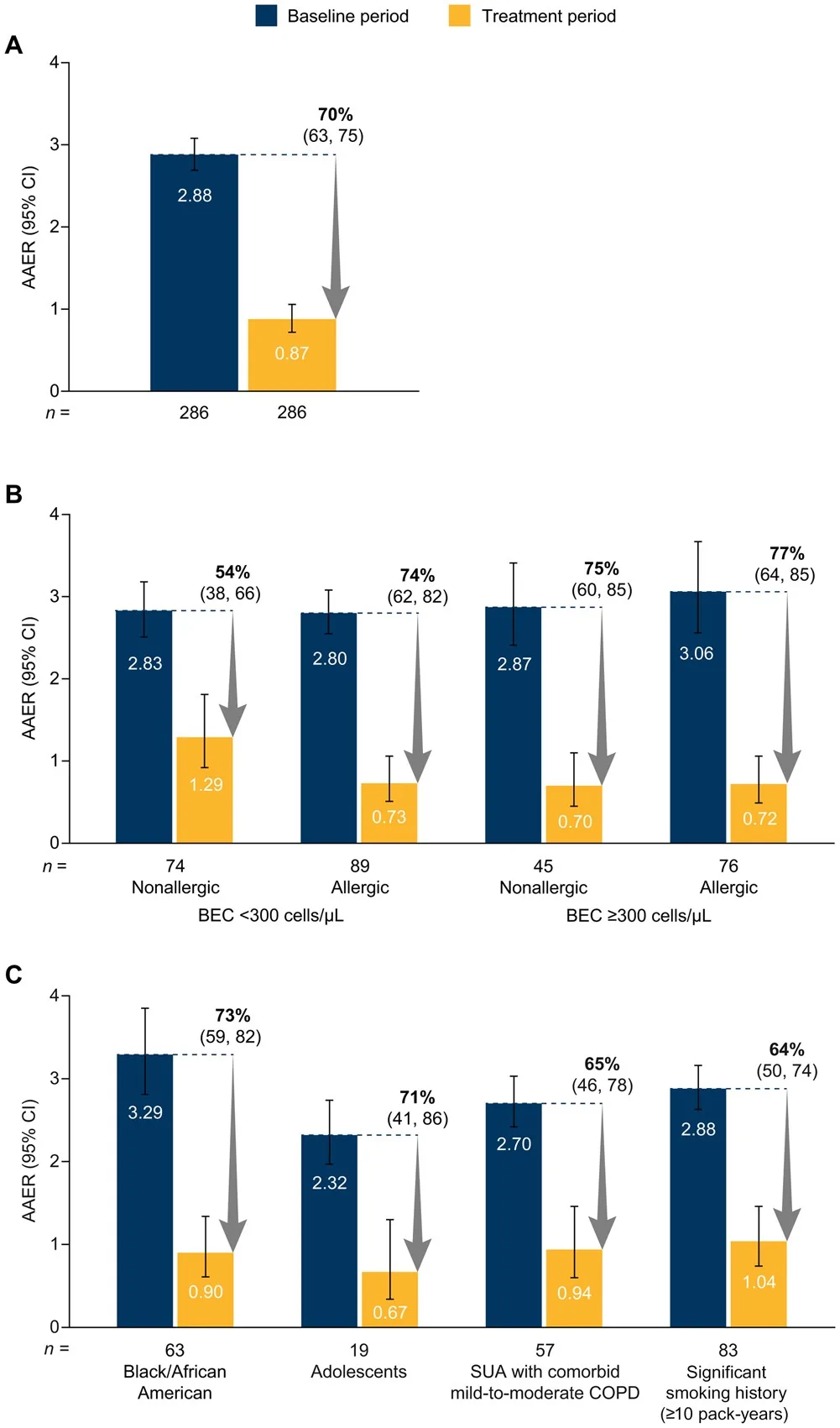
Reduction in the AAER from the baseline period to the treatment period in the overall population (A), in participants with nonallergic and allergic asthma according to BEC (B), and in underrepresented populations (C). Abbreviations: AAER, annualized asthma exacerbation rate; BEC, blood eosinophil count; CI, confidence interval; COPD, chronic obstructive pulmonary disease; SUA, severe, uncontrolled asthma.

### Outcomes

The primary outcome was the AAER in the 12-month treatment period relative to the 12-month baseline period (for further details, see the online supplement).

Secondary endpoints included assessments at baseline, week 24, and week 52 of pre-bronchodilator (BD) forced expiratory volume in 1 second (FEV_1_), asthma control (Asthma Control Questionnaire-6 [ACQ-6] score, Asthma Impairment and Risk Questionnaire [AIRQ] score), and HRQoL (St George’s Respiratory Questionnaire [SGRQ] total score). Exacerbations resulting in hospitalizations or ED/urgent care (UC) visits, cumulative systemic corticosteroid (SCS) dose, and asthma-related healthcare resource utilization (HCRU) were also assessed as secondary endpoints during the 12-month baseline and treatment periods.

Biomarker levels (BEC, total immunoglobulin E [IgE], and aeroallergen-specific IgE) were assessed at baseline and week 52. Fractional exhaled nitric oxide (FeNO) levels were collected if measured as part of routine clinical care. Exploratory endpoints included assessments at week 24 and week 52 of post-BD FEV_1_, FEV_1_ reversibility, Sino-Nasal Outcome Test-22 (SNOT-22) total score among participants with SUA with an ongoing, comorbid diagnosis of chronic rhinosinusitis at baseline (either without nasal polyps [CRSsNP] or with nasal polyps [CRSwNP]), Total Nasal Symptom Score (TNSS) among participants with severe nasal symptoms at baseline, proportion of frequent productive cough (FPC) responders, Patient Global Impression of Change (PGI-C) score among participants with SUA with a comorbid diagnosis of mild-to-moderate COPD, Clinical Global Impression of Change (CGI-C) score, and Treatment Satisfaction Questionnaire for Medication-9 (TSQM-9) score. Participant-reported causes of asthma symptoms or exacerbations were recorded in an asthma trigger survey conducted at baseline and week 52. A qualitative interview that examined participants’ asthma signs and symptoms before and after the study, as well as their experiences with tezepelumab during the study, was scheduled in the 3 months after the final study visit among 50 participants selected using targeted sampling to reflect the study’s demographic and clinical subgroup distribution. The proportions of participants meeting the study-specific definitions of clinical response and clinical remission were assessed at week 52.

Assessments of tezepelumab safety and tolerability included monitoring of serious adverse events (SAEs) and adverse events leading to discontinuation of tezepelumab during the treatment period.

### Ethical approval

The study was conducted in accordance with the Declaration of Helsinki, the International Council for Harmonisation Good Clinical Practice guidelines, and applicable regulatory requirements. Approvals from independent ethics committees were obtained for all study sites (Table S1). All participants provided written informed consent at the start of the study.

**Table 1:** Baseline demographics and clinical characteristics.

Characteristic	**Tezepelumab 210 mg Q4W (*N*** = **286)**
**Age, y**	
** Mean (SD)**	52.9 (16.2)
** Median (min, max)**	57 (12, 80)
**Female, No. (%)**	186 (65.0)
**Race, No. (%)**	
** White**	204 (71.3)
** Black/African American**	63 (22.0)
** Asian**	8 (2.8)
** Native Hawaiian or other Pacific Islander**	1 (0.3)
** American Indian or Alaska Native**	4 (1.4)
** Other**	4 (1.4)
** Not reported**	2 (0.7)
**Hispanic or Latino, No. (%)**	27 (9.4)
**Asthma duration, y, mean (SD)**	27.0 (20.4)
**Number of exacerbations in 12-month baseline period** ^a^ **, mean (SD)**	2.5 (1.3)
**Cumulative SCS dose during 12-month baseline period, mg, median (min, max) (*n*** = **279)**	560.0 (17.0, 8520.0)
**Prior asthma biologic use** ^b^ **, No. (%)**	3 (1)
**Smoking status, No. (%)**	
** Current**	22 (7.7)
** Former**	94 (32.9)
** Never**	170 (59.4)
**Pre-BD FEV_1_ (*n*** = **285)**	
** L, mean (SD)**	2.160 (0.735)
** L, median (Q1, Q3)**	2.060 (1.640, 2.510)
** Percent predicted, mean (SD)**	75.11 (20.17)
** Percent predicted ≤80%, No. (%; *n*** = **286)**	172 (60.1)
**Positive allergy status (perennial aeroallergen sensitization)** ^c^ **, No. (%)**	167 (58.4)
**Clinically relevant allergy to a perennial aeroallergen** ^d^ **, No. (%)**	120 (42.0)
**Number of asthma trigger types, median (Q1, Q3)**	11 (8, 12)
**Asthma triggers reported by ≥80% of participants, No. (%)**	
** Weather change**	253 (88.5)
** Viral infections**	241 (84.3)
** Exercise**	237 (82.9)
** Seasonal allergies**	232 (81.1)
**History of nasal polyps, No. (%)**	26 (9.1)
**SUA with ongoing diagnosis of CRSwNP, No. (%)**	25 (8.7)
**SUA with ongoing diagnosis of CRSsNP, No. (%)**	110 (38.5)
**BEC, cells/µL (*n*** = **284)**	
** Mean (SD)**	307 (312)
** ≥150, No. (%)**	180 (62.9)
** <150, No. (%)**	104 (36.4)
** ≥300, No. (%)**	121 (42.3)
** <300, No. (%)**	163 (57.0)
**Serum total IgE, IU/mL, mean (SD)** **Asthma phenotype** ^c^ ^,^ ^e^ **, No. (%; *n*** = **284)**	350 (676)
** Nonallergic asthma with BEC <300 cells/µL**	74 (25.9)
** Allergic asthma with BEC <300 cells/µL**	89 (31.1)
** Nonallergic asthma with BEC ≥300 cells/µL**	45 (15.7)
** Allergic asthma with BEC ≥300 cells/µL**	76 (26.6)
**Underrepresented populations, No. (%)**	
** Black/African American**	63 (22.0)
** Adolescent**	19 (6.6)
** SUA with comorbid mild-to-moderate COPD**	57 (19.9)
** People with a significant smoking history (≥10 pack-years)** ^f^	83 (29.0)
**Pre-BD FEV_1_ for participants with SUA with comorbid mild-to-moderate COPD (*n*** = **56)**	
** L, mean (SD)**	1.715 (0.558)
** L, median (Q1, Q3)**	1.640 (1.340, 1.990)
** Percent predicted, mean (SD)**	67.09 (20.98)
** Percent predicted ≤80%, No. (%)**	40 (70.2)
**BEC for participants with SUA with comorbid mild-to-moderate COPD, cells/µL (*n*** = **56)**	
** Mean (SD)**	231 (242)
** ≥150, No. (%)**	28 (49.1)
** <150, No. (%)**	28 (49.1)
** ≥300, No. (%)**	16 (28.1)
** <300, No. (%)**	40 (70.2)

Data are all *N* = 286 unless shown otherwise (one participant had missing data for pre-BD FEV_1_ and 2 participants had missing data for BEC; 279 participants had any SCS use during the baseline period).

Abbreviations: BD, bronchodilator; BEC, blood eosinophil count; COPD, chronic obstructive pulmonary disease; CRSsNP, chronic rhinosinusitis without nasal polyps; CRSwNP, chronic rhinosinusitis with nasal polyps; FEV_1_, forced expiratory volume in 1 second; Q1, quartile 1; Q3, quartile 3; Q4W, every 4 weeks; SCS, systemic corticosteroid; SD, standard deviation; SUA, severe, uncontrolled asthma.

a Twenty-four participants (8.4%) had one exacerbation and one participant (0.3%) had no exacerbations during the baseline period.

b Cessation of prior biologic treatment occurred more than 4 months or 5 half-lives (whichever was longer) before screening.

c Positive allergy status defined as a positive fluorescence enzyme immunoassay test result (≥0.35 kUA/L) for serum specific immunoglobulin E against a perennial aeroallergen at baseline.

d Defined as a clinical history of asthma symptoms and sensitization to a perennial aeroallergen at baseline.

e BEC group defined by the highest documented BEC in the previous year.

f Included people with a current or former smoking history.

### Statistical analyses

All reported analyses are descriptive in nature.

The AAER ratio (treatment period vs baseline period), including that for exacerbations resulting in hospitalization or ED/UC visits, was estimated using a generalized estimating equations model for repeated measures.

For continuous endpoints (other than biomarker levels), least-squares (LS) mean changes from baseline to weeks 24 and 52 were estimated using a model for repeated measures. Responders were defined as participants who achieved minimal clinically important difference thresholds for each outcome (≥5% or 0.1 L improvement from baseline for pre-BD FEV_1_; an absolute decrease from baseline of ≥0.5 points for ACQ-6, ≥2 points for AIRQ, ≥4 points for SGRQ total, ≥8.9 points for SNOT-22 total, and ≥3 points for FPC composite scores).^19-23^

Clinical response was defined as a reduction in asthma exacerbations of ≥50% between the baseline and treatment periods or a reduction in daily oral corticosteroid (OCS) use of ≥50%, a change from baseline in ACQ-6 score of ≥ −0.5, or a change in pre-BD FEV_1_ of ≥0.1 L at week 52. Clinical remission was defined as no exacerbations from week 4 after tezepelumab initiation and no daily OCS use, an ACQ-6 score of <1.5, and a ratio of post-treatment to baseline pre-BD FEV_1_ (in L) of ≥0.95 at week 52.

For further details on the statistical methodology, see the online supplement.

## Results

### Baseline demographics and clinical characteristics

Overall, 286 participants received at least one dose of tezepelumab and were included in the final analysis. Of these, 255 (89.2%) completed the study and 251 (87.8%) completed treatment (received the last dose in the study period) (Figure S2). The most common reasons for study withdrawal were withdrawal by participant (*n* = 16) and loss to follow-up (*n* = 6), and for treatment discontinuation were participant decision (*n* = 18) and adverse event (*n* = 8). The mean (standard deviation [SD]) treatment compliance to tezepelumab was 99.1% (3.4).

**Figure 2:**
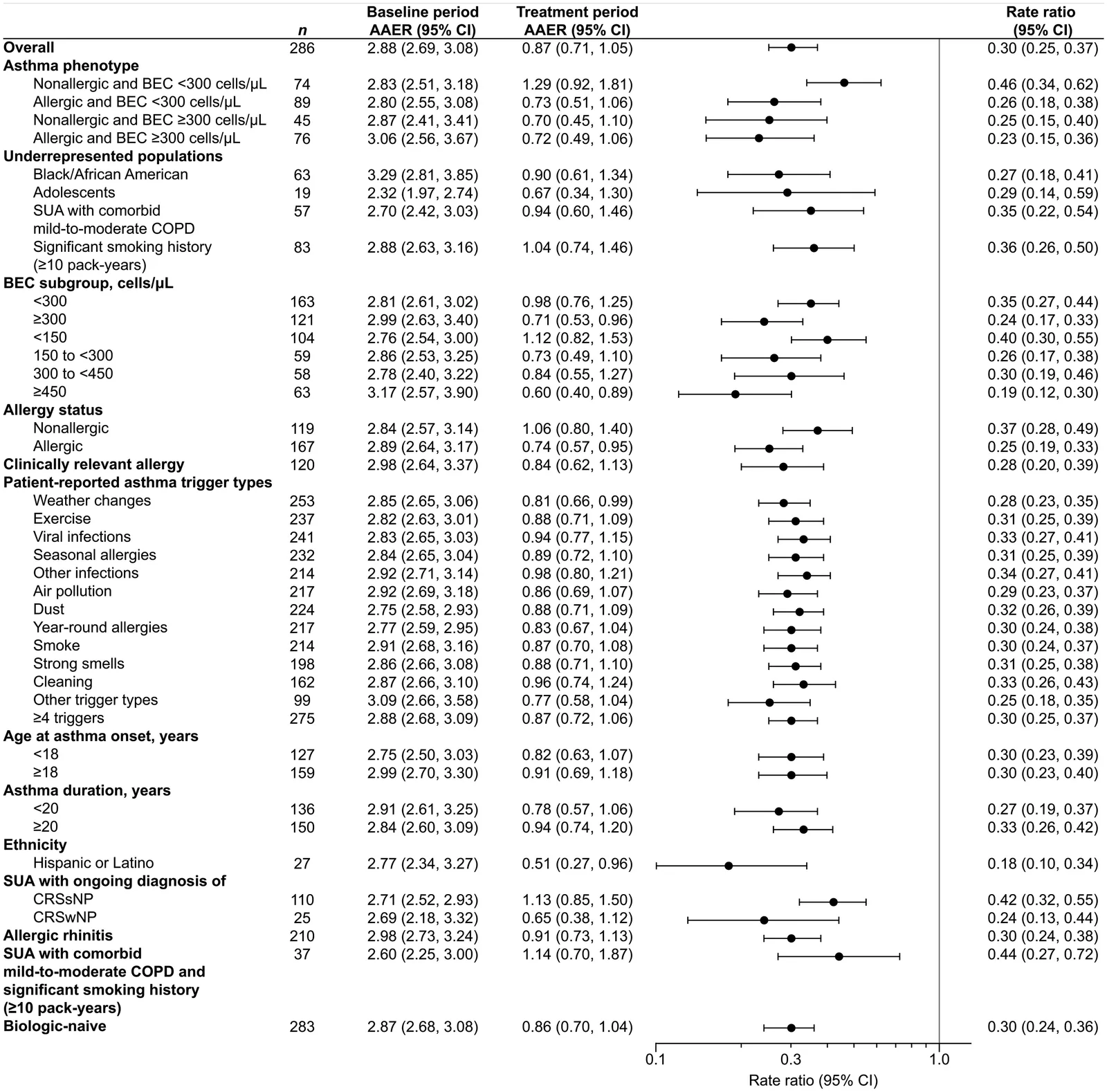
Rate ratios for reductions in the AAER from the baseline period to the treatment period in the overall population and in key patient subgroups. Abbreviations: AAER, annualized asthma exacerbation rate; BEC, blood eosinophil count; CI, confidence interval; COPD, chronic obstructive pulmonary disease; CRSsNP, chronic rhinosinusitis without nasal polyps; CRSwNP, chronic rhinosinusitis with nasal polyps; SUA, severe, uncontrolled asthma.

The mean (SD) age was 52.9 (16.2) years, and 65.0% of participants were female (Table 1). Regarding asthma phenotype, 25.9% and 31.1% of participants had nonallergic and allergic asthma with a BEC of <300 cells/µL, respectively, and 15.7% and 26.6% had nonallergic and allergic asthma with a BEC of ≥300 cells/µL, respectively. Regarding the 4 underrepresented populations targeted for enrollment, 22.0% of participants were Black/African American, 6.6% were adolescents, 19.9% had SUA with a comorbid diagnosis of mild-to-moderate COPD, and 29.0% had a significant smoking history. Additionally, 8.7% and 38.5% of participants had SUA with an ongoing diagnosis of CRSwNP and CRSsNP, respectively. The mean (SD) number of asthma exacerbations during the 12-month baseline period before tezepelumab initiation was 2.5 (1.3). At baseline, the mean (SD) pre-BD FEV_1_ was 2.160 L (0.74) and percent predicted FEV_1_ was 75.11% (20.17%); 60.1% of participants had a percent predicted pre-BD FEV_1_ of ≤80%.

### AAER

In the overall population, the AAER decreased by 70% (95% confidence interval [CI], 63%-75%; *N* = 286) from 2.88 before tezepelumab initiation (baseline period) to 0.87 after tezepelumab initiation (treatment period) (Figures 1A and 2). The relative reduction in the annualized rate of exacerbations resulting in hospitalization or an ED/UC visit from the baseline to treatment period was 76% (95% CI, 64%-84%; *N* = 286) in the overall population.

Across asthma phenotypes based on both allergy status and BEC, relative reductions in AAERs ranged from 54% in participants with nonallergic asthma and BEC <300 cells/µL to 77% in those with allergic asthma and BEC ≥300 cells/µL (Figures 1B and 2). In underrepresented populations, relative reductions in AAERs ranged from 64% in participants with a significant smoking history to 73% in Black/African American participants (Figures 1C and 2).

Considering BEC and allergy status separately, the relative reductions in AAERs were 65% and 76% in participants with BEC <300 cells/µL and ≥300 cells/µL, respectively, and 63% and 75% in participants with nonallergic and allergic asthma (sensitization to any perennial aeroallergen), respectively (Figure 2). The relative reduction in the AAER was 60% in participants with BEC <150 cells/µL and 72% in participants with evidence of clinically relevant perennial allergy (sensitization to and asthma symptoms from the same perennial aeroallergen). The reductions in AAERs in participants with SUA with an ongoing diagnosis of CRSsNP and CRSwNP were 58% and 76%, respectively.

### Spirometry

**Figure 3:**
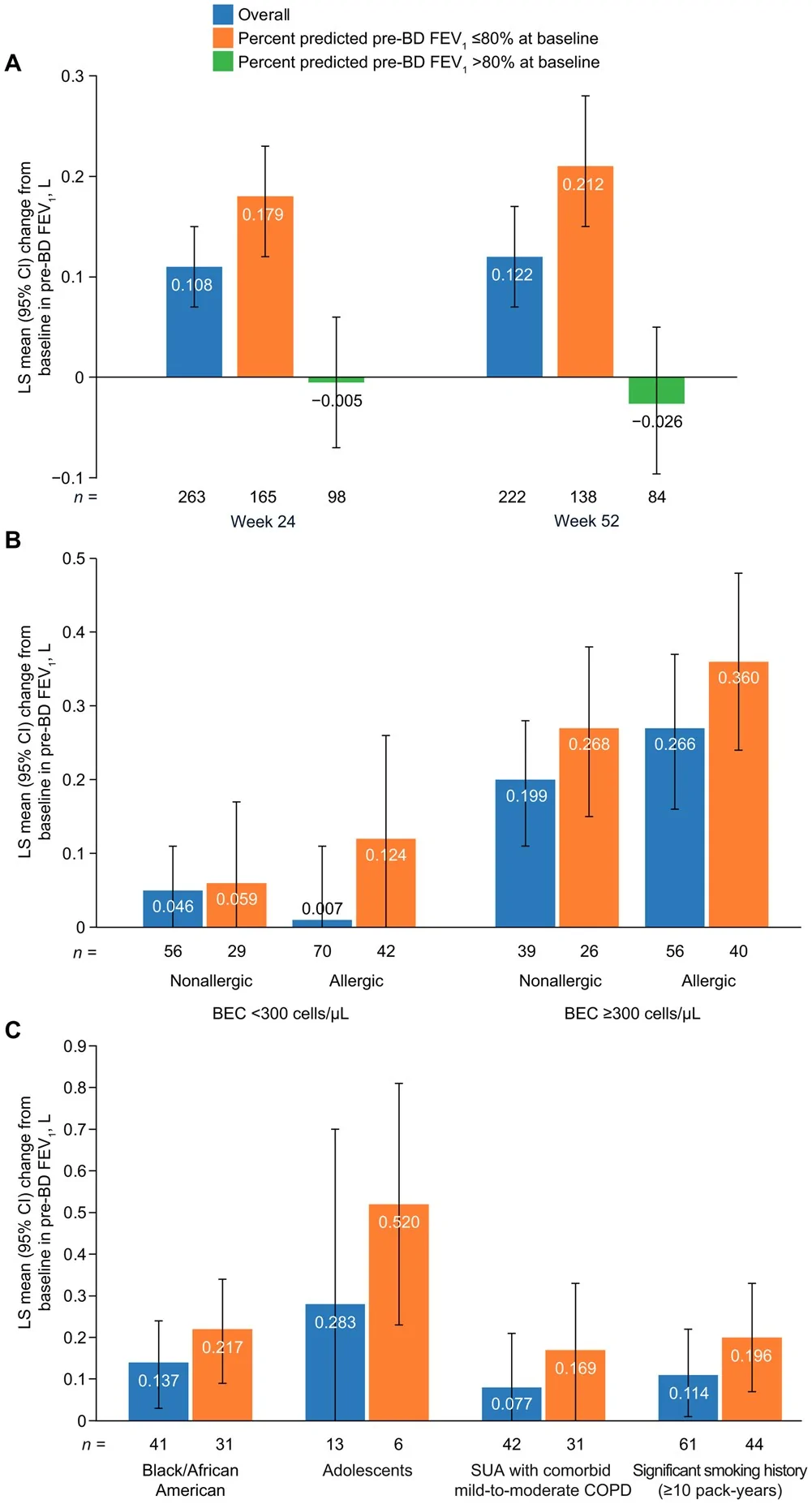
Improvements from baseline in pre-BD FEV_1_ in the overall population at weeks 24 and 52 (A), and across asthma phenotypes (B) and underrepresented populations (C) at week 52. Data for participants with percent predicted pre-BD FEV_1_ >80% at baseline were evaluated for the overall population only. Abbreviations: BD, bronchodilator; BEC, blood eosinophil count; CI, confidence interval; COPD, chronic obstructive pulmonary disease; FEV_1_, forced expiratory volume in 1 second; LS, least-squares; SUA, severe, uncontrolled asthma.

The LS mean change from baseline to week 52 in pre-BD FEV_1_ was 0.122 L (95% CI, 0.07-0.17; *n* = 222) in the overall population and 0.212 L (95% CI, 0.15-0.28; *n* = 138) and −0.026 L (95% CI, −0.10 to 0.05; *n* = 84) in participants with percent predicted pre-BD FEV_1_ ≤80% and >80% at baseline, respectively (Figure 3A). Across asthma phenotypes, increases in pre-BD FEV_1_ from baseline to week 52 ranged from 0.007 L to 0.266 L (Figure 3B). Among underrepresented populations, increases ranged from 0.077 L in participants with SUA with comorbid mild-to-moderate COPD to 0.283 L in adolescents (Figure 3C). The increase among participants with SUA with an ongoing diagnosis of CRSwNP was 0.128 L (95% CI, −0.07 to 0.33; *n* = 19).

The proportion of pre-BD FEV_1_ responders at week 52 among those with data was 48% (107/222) in the overall population, and 59% (81/138) and 23% (26/113) in participants with percent predicted pre-BD FEV_1_ ≤80% and >80% at baseline, respectively. The mean increase in post-BD FEV_1_ from baseline to week 52 was 0.087 L, and the mean decrease in FEV_1_ reversibility was 1.17% (Figure S3).

### Asthma control and HRQoL outcomes

**Figure 4:**
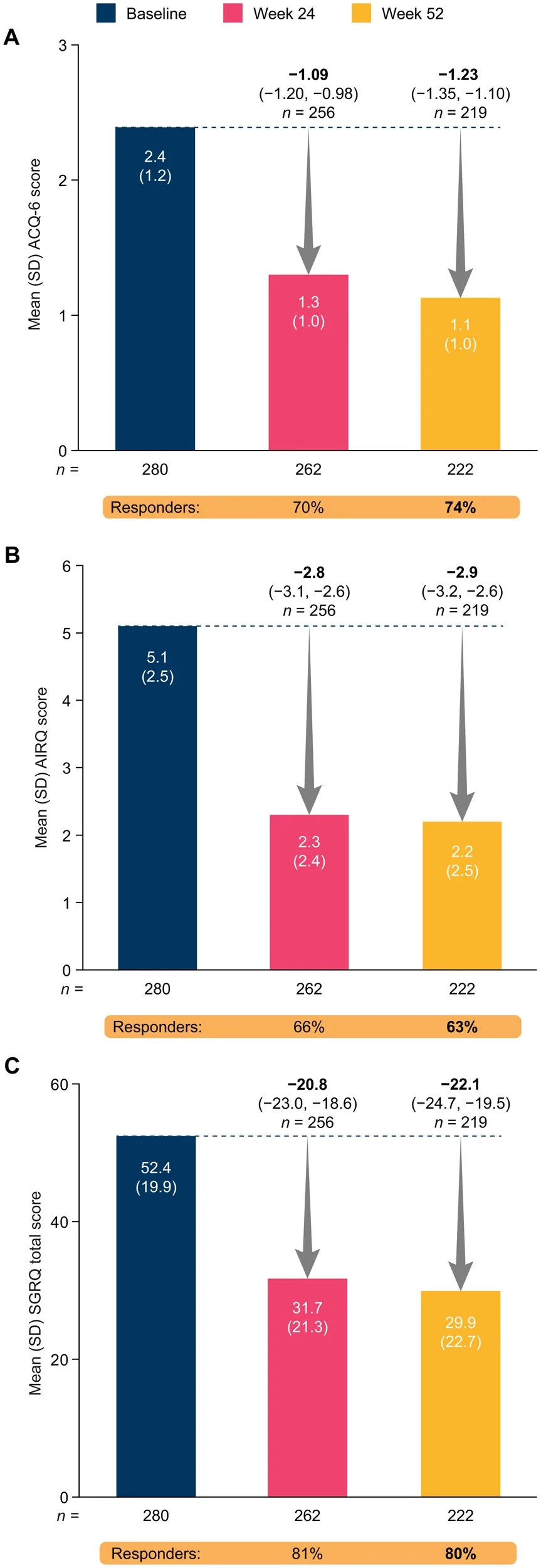
Improvements from baseline to weeks 24 and 52 in ACQ-6 score (A), AIRQ score (B), and SGRQ total score (C) in the overall population. The values above the bars are LS mean (95% CI) changes from baseline to weeks 24 and 52. Responders were defined as participants who achieved minimal clinically important difference thresholds for each outcome, ie, an absolute decrease from baseline of ≥0.5 points for ACQ-6 score, ≥2 points for AIRQ score, and ≥4 points for SGRQ total score. Abbreviations: ACQ-6, Asthma Control Questionnaire-6; AIRQ, Asthma Impairment and Risk Questionnaire; CI, confidence interval; LS, least-squares; SD, standard deviation; SGRQ, St George’s Respiratory Questionnaire.

At week 52, the LS mean change from baseline in ACQ-6 score was −1.23, and 74% of participants (162/219) were responders (Figure 4A). Among participants with ACQ-6 score ≥1.5 at baseline (*n* = 223, 77.9%; *n* = 175 with data at week 52), 64.6% of participants (113/175) had controlled asthma (ACQ-6 score <1.5) and 35.4% (62/175) had uncontrolled asthma (ACQ-6 score ≥1.5) at week 52. For AIRQ score, the LS mean change from baseline to week 52 was −2.9, and 63% of participants (139/219) were responders (Figure 4B). For SGRQ total score, the LS mean change from baseline to week 52 was −22.1, and 80% of participants (175/219) were responders (Figure 4C). The SGRQ domain with the largest reduction from baseline was symptoms (−27.4). Across asthma phenotypes and underrepresented populations, proportions of responders ranged from 64% to 86% for ACQ-6 score, from 51% to 75% for AIRQ score, and from 63% to 91% for SGRQ total score (Figure S4; Table S2).

### SCS use

The number of participants who required any SCS use was 279 (97.6%) during the baseline period and 129 (45.1%) during the treatment period. The median (min, max) cumulative SCS dose was 560.0 mg (17.0, 8520.0) in the baseline period (*n* = 279) and 350.0 mg (10.0, 19 907.5) in the treatment period (*n* = 129). There was a mean cumulative SCS dose reduction of 52.8% overall between the baseline and treatment periods (*n* = 279); the LS mean change from baseline to week 52 was −535.1 mg (95% CI, −690.7 to −379.3).

### Asthma-related HCRU

Participants in the overall population reduced their HCRU rates from the baseline to treatment periods in terms of ambulance transport (44%), telephone calls to a physician (26%), and telephone calls to a specialist (21%). Other HCRU rates were either comparable or modestly increased (Table S3).

### Biomarkers

The mean (SD) change from baseline to week 52 was −141 (286) cells/µL for BEC and −73.79 (339.74) IU/mL for serum total IgE level (Table S4). Among perennial aeroallergen-sensitized participants, serum allergen-specific IgE levels reduced from baseline to week 52 for all aeroallergens included in the analysis. Change in FeNO levels was not assessed owing to the small number of participants with baseline data (*n* = 17).

### Comorbid chronic rhinosinusitis symptoms and sinonasal-specific HRQoL

The LS mean change in SNOT-22 total score from baseline to week 52 was −12.9 in participants with SUA with comorbid CRSsNP (Figure S5A) and −16.1 in those with CRSwNP (Figure S5B). At week 52, the proportions of SNOT-22 responders among participants with SUA with comorbid CRSsNP and CRSwNP were 61% (42/69) and 53% (8/15), respectively. Among participants with any individual TNSS symptom scored severe at baseline, mean TNSS decreased by 4.3 to 6.5 across severe symptom subgroups from baseline to week 52 (Figure S5C).

### Comorbid COPD symptoms and response to treatment

Among participants with FPC at baseline, 75% of participants with SUA with comorbid mild-to-moderate COPD and 71% of those without comorbid mild-to-moderate COPD were FPC responders at week 52 (Figure S6A). Among participants with SUA with a comorbid diagnosis of mild-to-moderate COPD who completed the PGI-C at week 52, 58% of participants indicated that their asthma symptoms were much better following tezepelumab treatment and 16% said they were moderately better (Figure S6B).

### Clinical response and remission, physician-reported response, and treatment satisfaction

At the end of the 52-week treatment period, 99% of participants (283/286) met the criteria for clinical response in at least one domain and 37.8% (82/217) met the criteria for clinical remission. Clinicians determined via the CGI-C that overall asthma status was much improved in 41.8% (90/215) of participants and very much improved in 37.2% (80/215) of participants after 52 weeks of tezepelumab treatment (Figure S7A). Mean TSQM-9 domain scores at week 52 ranged from 76.4 to 77.2 (Figure S7B).

### Exit interview

All 51 invited participants completed an exit interview. Overall, 96.1% (*n* = 49) reported improvements in at least one symptom following treatment. Nearly all participants (98%) would recommend tezepelumab to other patients with asthma, and most (84%) said it met or exceeded their expectations.

### Safety

In total, 28 participants (9.8%) reported an SAE during the treatment period and 6 participants (2.1%) discontinued treatment owing to an SAE (Table S5). Infections and infestations were the most common class of SAEs, reported by 13 participants (4.5%) (Table S5). One death was reported, which was due to myocardial infarction; this was considered unrelated to study treatment. The primary cause of death was arteriosclerotic cardiovascular disease, and the secondary cause was hypertension.

## Discussion

PASSAGE provides the first prospective, post-authorization study of tezepelumab in a diverse, real-world US population with SUA, with a population intentionally enriched for patients with different asthma phenotypes and those who have historically been underrepresented or excluded from RCTs. Across asthma phenotypes and underrepresented populations, participants with SUA treated with tezepelumab demonstrated substantial reductions in asthma exacerbations. There were also clinically meaningful improvements in lung function, asthma control, and HRQoL, with no new safety signals identified. Results were consistent with the phase 3 NAVIGATOR study.^14^

Across inflammatory phenotypes, tezepelumab demonstrated effectiveness consistent with its pivotal clinical trials.^13^^,^^14^ AAER reductions spanned 54% to 77% across subgroups defined jointly by BEC and allergy status. The numerically largest relative reductions were apparent in type 2–associated endotypes (76% for participants with baseline BEC ≥300 cells/µL and 75% for participants with allergic asthma), yet clinically important reductions also occurred in lower type 2 profiles, including a 60% reduction in participants with BEC <150 cells/µL. These findings mirror those of a pooled analysis of PATHWAY and NAVIGATOR, in which tezepelumab reduced exacerbations across BEC strata and allergy statuses, with numerically greater effects in type 2-high disease, while also showing benefits in other phenotypes.^24^ Increases in lung function (particularly in participants with baseline BEC ≥300 cells/µL) and clinically meaningful improvements in patient-reported outcomes accompanied the reduction in exacerbations.

Despite the disproportionate burden of severe asthma morbidity and mortality in Black/African American patients,^25^ their representation remains low in RCTs (comprising 3.5% and 5.8% of the PATHWAY and NAVIGATOR populations, respectively, for example).^13^^,^^14^ With 22% representation, PASSAGE provides much-needed real-world evidence for tezepelumab in Black/African American patients. This subgroup had the highest AAER during the baseline period (LS mean: 3.29) among the underrepresented populations and a 73% AAER reduction from the baseline to treatment periods, with improvements in asthma control and HRQoL comparable to the overall cohort.

Importantly, PASSAGE prospectively enrolled SUA patients with a comorbid diagnosis of mild-to-moderate COPD (GOLD etiotype “COPD and asthma”; COPD-A; comprising ∼20% of the population) and people with a significant smoking history (29%).^26^ These populations are commonly excluded from biologic trials (including from PATHWAY and NAVIGATOR) owing to concerns regarding fixed airflow obstruction and alternative disease mechanisms.^13^^,^^14^ Nevertheless, significant reductions in the AAER were observed in these populations, namely a 65% reduction among SUA patients with comorbid mild-to-moderate COPD and a 64% reduction in people with a significant smoking history. Lung function improvements were more modest in SUA patients with comorbid mild-to-moderate COPD than in the overall population (mean pre-BD FEV_1_ increase of 0.077 L vs 0.122 L), consistent with the fixed component of airflow limitation and with the observation that higher proportions of SUA patients with comorbid mild-to-moderate COPD than the overall population had baseline BECs of <150 and <300 cells/µL; however, exacerbation benefits were preserved.

Only 19 adolescents were enrolled in PASSAGE (comprising 6.6% of the study population), which limits the conclusions that can be drawn for this population. Adolescent participation was challenging owing to factors such as academic schedules and the need to attend study visits. The study team implemented numerous mitigation strategies to increase adolescent enrollment during the recruitment period, but recruitment remained slow and no viable options to expedite it were identified. To avoid further delays to the study, enrollment was closed even though the target number (*n* = 50) was not met. Notwithstanding the small sample size, the adolescent population had a 71% (95% CI, 41%-86%) reduction in the AAER between the baseline and treatment periods. Evidence for the effectiveness of biologic therapies in adolescents remains limited, and the PASSAGE findings complement existing clinical data supporting tezepelumab use in patients from 12 years of age.^14^

Additional improvements were observed following tezepelumab treatment. Participants requiring SCS at baseline reduced their cumulative SCS dose by over 50% at week 52. HCRU rates were generally reduced from baseline or were comparable between the baseline and treatment periods. Furthermore, ∼38% of participants met clinical remission criteria at week 52, having no exacerbations or maintenance OCS use in the prior 52 weeks together with improved asthma control and preserved lung function.

Typical of descriptive real-world evidence studies for approved therapies with known efficacy, PASSAGE had a single-arm design with no comparator group, meaning that results may be influenced by factors such as regression to the mean. Baseline exacerbations were extracted retrospectively from medical records and may have been misclassified. Analyses for adolescents were limited by the small sample size owing to challenges in recruiting adolescent patients, despite various efforts to enrich this population. Finally, PASSAGE was conducted in specialist US centers, which may limit generalizability to broader or non-US populations. Despite these considerations, study enrollment exceeded the target of 50 patients for most asthma phenotypes and underrepresented population groups. The data generated significantly extend the evidence base for tezepelumab by demonstrating robust, comprehensive effectiveness in a heterogeneous and clinically complex population reflective of real-world severe asthma, which included patients encountered frequently in specialist practice but rarely represented in RCTs.

## Conclusion

PASSAGE provides real-world evidence of the effectiveness of tezepelumab in a diverse population of patients with SUA in the United States. Across asthma phenotypes and multiple populations that have historically been underrepresented in RCTs, tezepelumab recipients had substantial reductions in asthma exacerbations. There were also clinically meaningful improvements in lung function, asthma control, and HRQoL at 52 weeks post-initiation.

## Supplementary Material

aamag225_Supplementary_Data

## Data Availability

This study is registered at ClinicalTrials.gov with the identifier NCT05329194 (registration date: March 17, 2022). Data underlying the findings described in this article may be obtained in accordance with AstraZeneca’s data sharing policy described at: https://astrazenecagrouptrials.pharmacm.com/ST/Submission/Disclosure. Data for studies directly listed on Vivli can be requested through Vivli at www.vivli.org. Data for studies not listed on Vivli can be requested through Vivli at https://vivli.org/members/enquiries-about-studies-not-listed-on-the-vivli-platform/. AstraZeneca’s Vivli member page is also available, outlining further details: https://vivli.org/ourmember/astrazeneca/.
