# Supplementary material for: Tezepelumab in real-world US patients with severe asthma across phenotypes and underrepresented populations: the phase 4 PASSAGE study
Source: Am J Respir Crit Care Med. 2026 May 18;212(9):1988–98. doi: 10.1093/ajrccm/aamag225 (PMC13519350; doi:10.1093/ajrccm/aamag225)
Supplement: Supplementary file 1 [file aamag225_supplementary_data.zip › coi_disclosure_PA.docx]

| ICMJE DISCLOSURE FORM | |
| --- | --- |
| **Date:** | 12/3/2026 |
| **Your Name:** | Praveen Akuthota |
| **Manuscript Title:** | **Tezepelumab in Real-World U.S. Patients with Severe Asthma Across Phenotypes and Underrepresented Populations: The Phase 4 PASSAGE Study** |
| **Manuscript Number (if known):** | N/A |
| In the interest of transparency, we ask you to disclose all relationships/activities/interests listed below that are related to the content of your manuscript. “Related” means any relation with for-profit or not-for-profit third parties whose interests may be affected by the content of the manuscript. Disclosure represents a commitment to transparency and does not necessarily indicate a bias. If you are in doubt about whether to list a relationship/activity/interest, it is preferable that you do so.  The author’s relationships/activities/interests should be defined broadly. For example, if your manuscript pertains to the epidemiology of hypertension, you should declare all relationships with manufacturers of antihypertensive medication, even if that medication is not mentioned in the manuscript.  In item #1 below, report all support for the work reported in this manuscript without time limit. For all other items, the time frame for disclosure is the past 36 months. | |

|  | | | **Name all entities with whom you have this relationship or indicate none (add rows as needed)** | **Specifications/Comments (e.g., if payments were made to you or to your institution)** |
| --- | --- | --- | --- | --- |
| **Time frame: Since the initial planning of the work** | | | | |
| **1** | All support for the present manuscript (e.g., funding, provision of study materials, medical writing, article processing charges, etc.)  **No time limit for this item.** | | ☐ \| **None** Amgen Inc. \| Study funding; medical writing support; AstraZeneca \| Study funding; medical writing support; \| Click the tab key to add additional rows. | |
| **Time frame: past 36 months** | | | | |
| **2** | | Grants or contracts from any entity (if not indicated in item #1 above). | ☐ \| **None** AstraZeneca \| Research support; Sanofi \| Research support; Recode Therapeutics \| Research support; Cystic Fibrosis Foundation \| Research support; National Institutes of Health (USA) \| Research support; Advanced Research Projects Agency for Health (USA) \| Research support; 4D Molecular Therapeutics \| Research support | |
| **3** | | Royalties or licenses | ☐ \| **None** UpToDate \| Received royalties | |
| **4** | | Consulting fees | ☐ \| **None** Amgen \| Consulting fees; AstraZeneca \| Consulting fees; Connect Biopharma \| Consulting fees; Enveda \| Consulting fees; Gilead \| Consulting fees; GSK \| Consulting fees; Sanofi \| Consulting fees; Vida Ventures \| Consulting fees; Regeneron Pharmaceuticals \| Consulting fees | |
| **5** | | Payment or honoraria for lectures, presentations, speakers bureaus, manuscript writing or educational events | ☐ \| **None** Advancing Knowledge in Healthcare \| Received fees for continuing medical education presentations; Medlive \| Received fees for continuing medical education presentations; MJH Life Sciences \| Received fees for continuing medical education presentations; PeerView \| Received fees for continuing medical education presentations; PRIME Continuing Medical Education \| Received fees for continuing medical education presentations; Rockpointe \| Received fees for continuing medical education presentations; Vindico Medical Education \| Received fees for continuing medical education presentations; Taiwan Society of COPD \| Received fees for presentations | |
| **6** | | Payment for expert testimony | ☐ \| **None** | |
| **7** | | Support for attending meetings and/or travel | ☐ \| **None** | |
| **8** | | Patents planned, issued or pending | ☐ \| **None** | |
| **9** | | Participation on a Data Safety Monitoring Board or Advisory Board | ☐ \| **None** Sandia National Laboratories \| Received fees for participating in data safety monitoring board | |
| **10** | | Leadership or fiduciary role in other board, society, committee or advocacy group, paid or unpaid | ☐ \| **None** | |
| **11** | | Stock or stock options | ☐ \| **None** | |
| **12** | | Receipt of equipment, materials, drugs, medical writing, gifts or other services | ☐ \| **None** | |
| **13** | | Other financial or non-financial interests | ☐ \| **None** | |
|  | |  |  | |
| **Please place an “X” next to the following statement to indicate your agreement:** | | | | |
| ☒ | | I certify that I have answered every question and have not altered the wording of any of the questions on this form. | | |
