# Supplementary material for: Tezepelumab in real-world US patients with severe asthma across phenotypes and underrepresented populations: the phase 4 PASSAGE study
Source: Am J Respir Crit Care Med. 2026 May 18;212(9):1988–98. doi: 10.1093/ajrccm/aamag225 (PMC13519350; doi:10.1093/ajrccm/aamag225)
Supplement: Supplementary file 1 [file aamag225_supplementary_data.zip › PASSAGE final results_revised_supplement_update_CLEAN_26May26.docx]

**Online Data Supplement**

**Tezepelumab in Real-World U.S. Patients with Severe Asthma Across Phenotypes and Underrepresented Populations: The Phase 4 PASSAGE Study**

Njira L. Lugogo, Praveen Akuthota, Kaharu Sumino, Autumn F. Burnette, Sameer K. Mathur, Andrew W. Lindsley, Jean-Pierre Llanos, Claudio Marchese, Christopher S. Ambrose, and Benjamin Emmanuel

Contents

[Supplementary Methods	4](#_Toc230691625)

[Inclusion Criteria	4](#_Toc230691626)

[Exclusion Criteria	5](#_Toc230691627)

[Allergy Definitions and Asthma Trigger Survey	6](#_Toc230691628)

[Statistical Analyses	7](#_Toc230691629)

[Primary and Secondary Endpoint Analyses	8](#_Toc230691630)

[Exploratory Endpoint Analyses	10](#_Toc230691631)

[Supplementary References	12](#_Toc230691632)

[Table S1. List of Ethics Committees and/or IRBs	13](#_Toc230691633)

[Table S2. Change from Baseline to Week 52 in Asthma Control and HRQoL Outcomes, Overall, by Asthma Phenotype and in Underrepresented Populations	16](#_Toc230691634)

[Table S3. Asthma-Related HCRU Annual Rates and Comparison from the Baseline Period to the Treatment Period in the Overall Population	18](#_Toc230691635)

[Table S4. Change from Baseline to Week 52 in BECs, Total IgE Levels, and Specific IgE Levels to Perennial Aeroallergens	20](#_Toc230691636)

[Table S5. Summary of SAEs Reported During the On-Treatment Period	21](#_Toc230691637)

[Figure S1. PASSAGE study design.	23](#_Toc230691638)

[Figure S2. Patient disposition.	23](#_Toc230691639)

[Figure S3. Change from baseline to week 52 in pre- and post-BD FEV_1_ (*A*) and in FEV_1_ reversibility (*B*) in the overall population.	24](#_Toc230691640)

[Figure S4. Proportion of responders at week 52 for ACQ-6 (*A*), AIRQ (*B*), and SGRQ total (*C*) scores in the overall population, by asthma phenotype and in underrepresented populations.	26](#_Toc230691641)

[Figure S5. Change from baseline to week 52 in SNOT-22 total score in participants with SUA with comorbid CRSsNP (*A*) and CRSwNP (*B*), and in TNSS among participants with SUA and a TNSS result indicating a severe symptom at baseline (*C*).	27](#_Toc230691642)

[Figure S6. Proportion of FPC responders at week 52 among participants with FPC at baseline (overall population, participants with SUA without comorbid mild-to-moderate COPD, participants with SUA with comorbid mild-to-moderate COPD) (*A*), and PGI-C response categories at week 52 among participants with SUA with comorbid mild-to-moderate COPD (*B*).	29](#_Toc230691643)

[Figure S7. CGI-C response categories (*A*) and mean TSQM-9 domain scores (*B*) at week 52 in the overall population.	31](#_Toc230691644)

# Supplementary Methods

## Inclusion Criteria

Participants were eligible to be included in the study only if they met all of the following criteria.

1. Male or female participant 12 years of age or older at the time of signing the informed consent form or providing assent.
2. Documented physician-diagnosed asthma for at least 12 months before the first visit and confirmed by the investigator not to be due to alternative diagnoses.
3. Documented treatment with medium- to high-dose inhaled corticosteroids (ICS) as per Global Initiative for Asthma guidelines [S1] for at least 12 months before the first visit.
4. Use of additional asthma maintenance controller medication(s) in addition to ICS (e.g., long-acting β2 agonists [LABAs], leukotriene receptor inhibitors, theophylline, long-acting muscarinic agents, and cromones) for at least 12 months before the first visit. The additional maintenance controller medication could have been contained in a combination product (e.g., ICS/LABA).
5. Documented history of at least two asthma exacerbations during the 12 months before the first visit. Asthma exacerbations were defined as follows.

- A temporary bolus/burst of systemic corticosteroids (SCS; or a temporary increase in stable oral corticosteroid [OCS] background dose) for at least 3 consecutive days to treat symptoms of asthma worsening; a single depo-injectable dose of corticosteroids was considered equivalent to a 3-day bolus/burst of SCS.
- An emergency department (ED) or urgent care (UC) visit (defined as evaluation and treatment for <24 hours in an ED/UC center) owing to asthma that required SCS (as per the above).
- An inpatient hospitalization (defined as admission to an inpatient facility and/or evaluation and treatment in a healthcare facility for ≥24 hours) owing to asthma.

6. Physician decision that the participant was eligible for treatment with tezepelumab according to the approved U.S. prescribing information [S2].
7. Currently receiving care from specialist physicians (e.g., pulmonologists and/or allergists) at the investigator or sub-investigator’s site.
8. Provision of signed and dated written informed consent before any mandatory study-specific procedures, sampling, and analyses for participants who were the age of majority or older (as per local law). For participants who were younger than the age of majority, in addition to providing informed assent, their legally authorized representative was required to provide their informed consent.

## Exclusion Criteria

Participants were excluded from the study if any of the following criteria applied.

1. Any contraindication to tezepelumab as per the U.S. prescribing information [S2] or in the opinion of the investigator.
2. Comorbid diagnosis of severe or very severe COPD per Global Initiative for Chronic Obstructive Lung Disease (GOLD) guidelines [S3].
3. Use of biologics that are approved for the treatment of asthma in the 4 months or 5 half-lives (whichever was longer) before the first visit.
4. Participation in an interventional clinical trial for asthma in the 12 months before the first visit.
5. Judgment by the investigator that the participant was unlikely to adhere to study procedures, restrictions, and requirements.

## Allergy Definitions and Asthma Trigger Survey

Allergy status was determined according to perennial aeroallergen sensitization, which was defined as a positive fluorescence enzyme immunoassay (FEIA) test result (specific immunoglobulin E [IgE] ≥0.35 kUA/L) at baseline for IgE specific to any common perennial aeroallergen (cat dander, dog dander, American, oriental, or German cockroach [*Periplaneta americana*, *Blatta orientalis*, or *Blattella germanica*], mouse urine, dust mite [*Dermatophagoides farinae*, *Dermatophagoides pteronyssinus*], and molds/fungi [*Alternaria*, *Aspergillus fumigatus*, *Aspergillus niger*, *Cladosporium*, *Penicillium notatum*]).

Clinically relevant perennial allergy was defined as a clinical history of asthma symptoms driven by exposure to a perennial aeroallergen and sensitization to the corresponding perennial aeroallergen as determined by a positive FEIA test result (as above).

Patient-reported asthma triggers were assessed using the asthma trigger survey, which was a one-question survey conducted to collect data on the activities and/or other factors that trigger asthma symptoms and/or exacerbations at baseline. Participants could have had more than one reported asthma trigger.

## Statistical Analyses

All reported analyses are descriptive in nature; no formal hypotheses were tested statistically.

There was no predefined study hypothesis to be tested in this study. Because the study was descriptive in nature, all analyses followed an estimation‑based approach. A planned sample size of 400 participants was proposed based on practical considerations, including the need to enroll sufficient numbers across the pre-specified asthma phenotypes and to ensure approximately 50 or more participants in each population of interest: individuals who identified as Black/African American, adolescents, SUA patients with a comorbid diagnosis of mild‑to‑moderate COPD, and participants with a significant smoking history.

Statistical power to detect a significant difference between exacerbation rates in the baseline and treatment periods was evaluated using hypothetical true rate ratios and sample sizes in a two‑group comparison of incidence rates based on a negative binomial model. This calculation assumed a baseline rate of 2.25 asthma exacerbations per person‑year prior to tezepelumab treatment, a common dispersion parameter of 1.4, a significance level of 0.05, and a mean exposure duration of one year.

No imputation was used for missing data.

Percentage treatment compliance to tezepelumab was calculated as the total number of actual dosing occasions / the total number of expected dosing occasions × 100.

## Primary and Secondary Endpoint Analyses

An asthma exacerbation was defined as a worsening of asthma symptoms, characterized by use of SCS for at least 3 consecutive days, or an ED/UC visit owing to asthma that required use of SCS for at least 3 consecutive days, and/or inpatient hospitalization (≥24 hours) owing to asthma. Any asthma exacerbation that occurred within 7 days of a previous exacerbation was counted as the same exacerbation event. Records of asthma exacerbations were collected from medical records at week 0 for the baseline period and were recorded in an electronic case report form at weeks 24 and 52 for the treatment period.

The AAER ratio (treatment period vs. baseline period; primary endpoint) was estimated using a generalized estimating equations model for repeated measures with a log link function, in which the response variable (number of exacerbations) was modeled using a negative binomial distribution and with period (baseline period, treatment period) and age (continuous) as covariates. The logarithm of the time at risk for exacerbations was used as an offset variable. AAER ratios were calculated as the ratio of the AAER in the treatment period to the baseline period (ratios <1 indicate a reduction in the AAER in the treatment period compared with the baseline period). Percentage changes in AAER between the baseline and treatment periods were calculated as (1 − rate ratio) × 100. All data collected during the treatment period were included in the study analysis up to the date of study completion or participant withdrawal.

For secondary endpoints, baseline values were the last recorded assessment on or before the first dose of tezepelumab at week 0.

For pre-BD FEV_1_, ACQ-6 score, AIRQ score, and SGRQ total score, least-squares mean changes from baseline to weeks 24 and 52 were estimated using a model for repeated measures, in which the response variable was the change from baseline to week 24 or 52, including baseline value, age, and visit as covariates. The proportions of responders at weeks 24 and 52 were calculated and summarized descriptively among those with available data. Responders were defined as participants who achieved minimal clinically important difference thresholds for each outcome (≥5% or 0.1 L improvement from baseline for pre-BD FEV_1_; an absolute decrease from baseline of ≥0.5 points for ACQ-6, ≥2 points for AIRQ, ≥4 points for SGRQ total).

For cumulative SCS dose, the least-squares mean change from the baseline period to the treatment period was estimated using a model for repeated measures, including baseline cumulative SCS dose, age, and visit as covariates.

Healthcare resource utilization annual rates were estimated using a generalized estimating equation model for repeated measures using a log link function, in which the number of healthcare resource utilization events following a negative binomial distribution was included as the response variable, the logarithm of the follow-up time was used as an offset variable, and period (baseline period, treatment period) and age (continuous) were included as covariates. Comparisons between the baseline and treatment periods are presented as rate ratios.

Serious adverse events and adverse events leading to the discontinuation of tezepelumab were presented descriptively by system organ class and preferred term during the on-treatment period. The on-treatment period started on the index date and ended on the earliest of the following: date of last dose of study intervention + 35 days; date of death; date of study withdrawal. The date of study withdrawal was the completion or discontinuation date recorded in the electronic case report form. Adverse events were coded using the Medical Dictionary for Regulatory Activities (MedDRA) version 28.1.

## Exploratory Endpoint Analyses

Post-bronchodilator (BD) forced expiratory volume in 1 second (FEV_1_) was assessed at baseline and at weeks 24 and 52. Absolute values and changes from baseline were summarized descriptively. Percent FEV_1_ reversibility was assessed at weeks 24 and 52, and was defined as ([Post-BD FEV_1_ – Pre-BD FEV_1_] / Pre-BD FEV_1_) × 100% for pre- and post-BD measurements taken on the same date.

Sino-Nasal Outcome Test-22 (SNOT-22) total score was assessed at weeks 24 and 52 in participants with SUA with an ongoing diagnosis of chronic rhinosinusitis at baseline. The least-squares mean change from baseline at each time point was estimated using a repeated measures analysis with baseline SNOT-22 total score, age, and visit as covariates. The proportions of responders at weeks 24 and 52, defined as achieving the minimal clinically important difference threshold of an absolute decrease from baseline of ≥8.9 points, were calculated and summarized descriptively among those with available data.

Total nasal symptom score (TNSS), which evaluates classic rhinitis symptoms, was assessed at baseline and at weeks 24 and 52. The TNSS questionnaire comprises four questions (one question per symptom) each with a score range of 0–3 (3 indicates a severe symptom), giving a maximum total score of 12. The analysis of TNSS scores was reported for those participants with any individual nasal symptom scored severe at baseline, grouped by symptom.

Frequent productive cough (FPC) was assessed at baseline and at weeks 24 and 52. FPC is a binary composite endpoint of two St George’s Respiratory Questionnaire (SGRQ) items: “Over the past 3 months, I have coughed…” and “Over the past 3 months, I have brought up phlegm (sputum)…”. FPC score is defined as the unweighted sum of the responses to the two SGRQ items. ‘Having FPC’ and ‘Not having FPC’ were defined as FPC composite scores of ≥3 and <3, respectively. FPC scores were analyzed descriptively by time point. The proportions of responders at weeks 24 and 52, defined as achieving the minimal clinically important difference threshold of an absolute decrease from baseline of ≥3 points in FPC composite score, were calculated and summarized descriptively among those with available data.

Patient Global Impression of Change (PGI-C) score for COPD was assessed at weeks 24 and 52 in participants with SUA with an ongoing comorbid diagnosis of COPD at baseline. PGI-C total scores were analyzed descriptively by time point.

Treatment Satisfaction Questionnaire for Medication-9 (TSQM-9) and Clinical Global Impression of Change (CGI-C) scores were assessed at weeks 24 and 52. TSQM-9 domain scores were calculated as the means of responses to the questions in each of the domains. TSQM-9 domain scores and CGI-C total score were analyzed descriptively by time point.

Clinical response was defined as a reduction in asthma exacerbations of ≥50% between the baseline and treatment periods, a reduction in daily OCS use of ≥50% at week 52, a change from baseline in ACQ-6 score of ≥−0.5, or a change in pre-BD FEV_1_ of ≥0.1 L. Clinical remission was defined as no exacerbations from week 4 after tezepelumab initiation, no daily OCS use at week 52, an ACQ-6 score of <1.5, and a ratio of post-treatment to baseline pre-BD FEV_1_ (in L) of ≥0.95.

All participants who completed or discontinued the study were eligible to participate in a voluntary virtual 45-minute interview with a trained interviewer in the 3 months after their final study visit. In total, 51 participants were selected using targeted sampling to reflect the demographic and clinical subgroup distribution in PASSAGE. The interviews surveyed participants’ asthma signs, symptoms, and health-related quality of life before and after PASSAGE, and participants’ experiences with tezepelumab during the study.

## Supplementary References

S1. Global Initiative for Asthma. Global Strategy for Asthma Management and Prevention. Available at: https://ginasthma.org/. Accessed December 12, 2024.
S2. TEZSPIRE™ (tezepelumab) prescribing information 2021. AstraZeneca. Available at: https://www.accessdata.fda.gov/drugsatfda_docs/label/2023/761224s001lbl.pdf. Accessed December 12, 2024.
S3. Global Initiative for Chronic Obstructive Lung Disease. Global strategy for the diagnosis, management, and prevention of chronic obstructive pulmonary disease: 2024 report. Available at: https://goldcopd.org/2024-gold-report/. Accessed December 12, 2024.

# Table S1. List of Ethics Committees and/or IRBs

| **IRB number** | **IRB name** | **City** | **State/country** | **IRB type** |
| --- | --- | --- | --- | --- |
| IRB00000971 | Advarra  (Central IRB)^a^ | Columbia (USA); Aurora (Canada) | MD, USA;  ON, Canada | OHRP/FDA |
| IRB00000428 | New York Medical College IRB #1 | Valhalla | NY, USA | OHRP/FDA |
| IRB00002528 | New York Medical College IRB #2 | Valhalla | NY, USA | OHRP/FDA |
| IRB00000244 | University of Michigan IRBMED A-1 #1 | Ann Arbor | MI, USA | OHRP/FDA |
| IRB00000245 | University of Michigan IRB #2 – Health Sciences and Behavioral Sciences Maize | Ann Arbor | MI, USA | OHRP only |
| IRB00000246 | University of Michigan IRB #3 – Health Sciences and Behavioral Sciences Blue | Ann Arbor | MI, USA | OHRP only |
| IRB00001995 | University of Michigan IRBMED B-2 #6 | Ann Arbor | MI, USA | OHRP/FDA |
| IRB00001996 | University of Michigan IRBMED A-2 #7 | Ann Arbor | MI, USA | OHRP/FDA |
| IRB00001999 | University of Michigan IRBMED B-1 #8 | Ann Arbor | MI, USA | OHRP/FDA |
| IRB00005467 | University of Michigan IRB #1 – IRBMED C-1 #9 | Ann Arbor | MI, USA | OHRP/FDA |
| IRB00012211 | University of Michigan  IRB #11 – IRBMED C-2 #11 | Ann Arbor | MI, USA | OHRP/FDA |
| IRB00002040 | Eisenhower Medical Center IRB #1 | Rancho Mirage | CA, USA | OHRP/FDA |

^a^The Advarra central IRB covers all study sites (see list below), except Children's and Women's Physicians of Westchester, LLP (covered by New York Medical College, Valhalla, NY), University of Michigan, Michigan Medicine (covered by University of Michigan, Ann Arbor, MI), Pulmonary Clinic and Eisenhower Health Center (covered by Rancho Mirage, CA).

Sites covered by the Advarra Central IRB included:

Clinical Research Associates of Central PA, LLC;

JPS Health Network;

Respiratory Medicine Research Institute of MI, PLC;

Allergy & Asthma Family Care of Queens, P.C.;

Temple University Health System – Temple Lung Center;

UNC at Chapel Hill – Adult Asthma Program;

Advanced Asthma Allergy & S.C.;

Allergy, Asthma & Immunology Assoc., Pc;

Methodist Physicians Clinic Westroads Office Park – Pulmonology;

Toledo Institute of Clinical Research Inc.;

American Institute of Research;

Allergy & Asthma Center of Minnesota – Allergology;

Yale University School of Medicine;

Washington University;

STAAMP Research;

The Vancouver Clinic;

University of Virginia;

Asthma and Allergy Assoc PC and Research Center;

Allianz Research Institute;

Allergy, Asthma and Clinical Research Center;

Allergy & Asthma Associates, PSC;

Metroplex Pulmonary and Sleep Center; ADAC Research, PA;

Northwestern Medical Group – Allergy & Immunology;

Tulane School of Medicine;

URMC Strong Memorial Hospital;

ARK Clinical Research, LLC;

Tennessee Comprehensive Lung & Sleep Center;

Accellacare.

*Definition of abbreviations:* FDA = US Food and Drug Administration; IRB = institutional review board; OHRP = Office for Human Research Protections

# Table S2. Change from Baseline to Week 52 in Asthma Control and HRQoL Outcomes, Overall, by Asthma Phenotype and in Underrepresented Populations

| **Outcome** | **Least-squares mean change from baseline to week 52 (95% CI)** |
| --- | --- |
| **ACQ-6 score** |  |
| Overall (n = 219) | −1.23 (−1.35, −1.10) |
| *Asthma phenotype^a^* |  |
| Allergic with BEC ≥ 300 (n = 55) | −1.29 (−1.59, −1.00) |
| Nonallergic with BEC ≥ 300 (n = 36) | −1.60 (−1.84, −1.36) |
| Allergic with BEC < 300 (n = 72) | −1.15 (−1.35, −0.95) |
| Nonallergic with BEC < 300 (n = 55) | −1.06 (−1.32, −0.80) |
| *Underrepresented populations* |  |
| Black/African American (n = 41) | −1.34 (−1.56, −1.12) |
| Adolescents (n = 11) | −1.58 (−2.11, −1.05) |
| SUA with comorbid mild-to-moderate COPD (n = 43) | −1.00 (−1.41, −0.60) |
| Significant smoking history  (≥10 pack-years)^b^ (n = 61) | −1.03 (−1.33, −0.72) |
| **AIRQ score** |  |
| Overall (n = 219) | −2.9 (−3.20, −2.60) |
| *Asthma phenotype^a^* |  |
| Allergic with BEC ≥ 300 (n = 55) | −2.8 (−3.29, −2.24) |
| Non-allergic with BEC ≥ 300 (n = 36) | −3.7 (−4.29, −3.21) |
| Allergic with BEC < 300 (n = 72) | −3.0 (−3.55, −2.41) |
| Non-allergic with BEC < 300 (n = 55) | −2.4 (−3.08, −1.76) |
| *Underrepresented populations* |  |
| Black/African American (n = 41) | −3.1 (−3.76, −2.50) |
| Adolescents (n = 11) | −3.8 (−4.53, −3.01) |
| SUA with comorbid mild-to-moderate COPD (n = 43) | −2.3 (−3.11, −1.55) |
| Significant smoking history  (≥10 pack-years)^b^ (n = 61) | −2.6 (−3.26, −2.01) |
| **SGRQ total score** |  |
| Overall (n = 219) | −22.12 (−24.71, −19.53) |
| *Asthma phenotype^a^* |  |
| Allergic with BEC ≥ 300 (n = 55) | −23.93 (−28.36, −19.49) |
| Non-allergic with BEC ≥ 300 (n = 36) | −26.04 (−32.15, −19.94) |
| Allergic with BEC < 300 (n = 72) | −21.53 (−26.24, −16.82) |
| Non-allergic with BEC < 300 (n = 55) | −19.25 (−25.09, −13.40) |
| *Underrepresented populations* |  |
| Black/African American (n = 41) | −19.03 (−24.35, −13.70) |
| Adolescents (n = 11) | −28.91 (−36.71, −21.11) |
| SUA with comorbid mild-to-moderate COPD (n = 43) | −14.02 (−20.54, −7.50) |
| Significant smoking history  (≥10 pack-years)^b^ (n = 61) | −17.58 (−23.35, −11.80) |

*Definition of abbreviations:* ACQ-6 = Asthma Control Questionnaire-6; AIRQ = Asthma Impairment and Risk Questionnaire; BEC = blood eosinophil count; CI = confidence interval; COPD = chronic obstructive pulmonary disease; FEIA = fluorescence enzyme immunoassay; IgE = immunoglobulin E; SGRQ = St George’s Respiratory Questionnaire; SUA = severe, uncontrolled asthma.

^a^Defined by allergy status (defined as a positive FEIA test result [≥ 0.35 kUA/L] for serum-specific IgE against a perennial allergen at baseline) and the highest documented BEC in the previous year.

^b^Included current and former smokers.

# Table S3. Asthma-Related HCRU Annual Rates and Comparison from the Baseline Period to the Treatment Period in the Overall Population

| **HCRU Event** | **HCRU Annual Rate, Estimate (95% CI)** | | **Comparison with Baseline Period, Rate Ratio (95% CI)** |
| --- | --- | --- | --- |
|  | **Baseline Period** | **Treatment Period** |  |
| Ambulance transport, *n* = 10 | 3.02 (1.80, 5.08) | 1.69 (0.74, 3.84) | 0.56 (0.21, 1.48) |
| Hospitalization intensive care, n = 12 | NE | NE | NE |
| UC visits, *n* = 93 | NE | NE | NE |
| ED visits, *n* = 89 | NE | NE | NE |
| Hospital admission or ED visit >24 hours, *n* = 49 | 2.16 (1.68, 2.76) | 2.10 (1.53, 2.89) | 0.97 (0.70, 1.36) |
| Visits to specialist, *n* = 234 | 3.08 (2.74, 3.47) | 3.54 (3.10, 4.04) | 1.15 (0.97, 1.36) |
| Visits to primary care physician, *n* = 139 | 2.97 (2.46, 3.58) | 3.08 (2.44, 3.88) | 1.04 (0.76, 1.42) |
| Other healthcare visits, *n* = 50 | NE | NE | NE |
| Telephone calls to physician, *n* = 59 | 4.37 (2.48, 7.69) | 3.22 (2.41, 4.29) | 0.74 (0.44, 1.24) |
| Telephone calls to nurse, *n* = 42 | 2.16 (1.60, 2.90) | 2.15 (1.49, 3.11) | 1.00 (0.63, 1.58) |
| Telephone calls to specialist, *n* = 103 | 3.44 (2.22, 5.33) | 2.72 (2.15, 3.44) | 0.79 (0.52, 1.21) |
| Telephone contacts other healthcare provider, *n* = 30 | NE | NE | NE |
| Spirometry assessments, *n* = 156 | 1.68 (1.43, 1.98) | 1.71 (1.38, 2.12) | 1.02 (0.78, 1.33) |
| Plain chest X-ray assessments, *n* = 108 | 1.89 (1.48, 2.39) | 1.90 (1.39, 2.59) | 1.01 (0.65, 1.54) |
| CT assessment, *n* = 71 | 1.59 (1.23, 2.05) | 1.59 (1.21, 2.07) | 1.00 (0.68, 1.47) |
| Number of days of hospitalization, *n* = 46 | NE | NE | NE |

*Definition of abbreviations:* CI = confidence interval; CT = computed tomography; ED = emergency department; HCRU = healthcare resource utilization; NE = not estimable owing to convergence issues; UC = urgent care.

# Table S4. Change from Baseline to Week 52 in BECs, Total IgE Levels, and Specific IgE Levels to Perennial Aeroallergens

| **Biomarker** | ***n*** | **Mean (SD) Change from Baseline to Week 52** |
| --- | --- | --- |
| BEC, cells/µL | 204 | −141 (286) |
| Total IgE, IU/mL | 188 | −73.79 (339.74) |
| *Alternaria alternata* IgE antibody, U/mL | 29 | −1.06 (3.68) |
| American cockroach antigen, U/mL | 6 | −1.49 (2.87) |
| *Aspergillus fumigatus* IgE antibody, U/mL | 33 | −2.86 (7.75) |
| Cat dander antigen IgE antibody, U/mL | 58 | −6.27 (12.99) |
| *Cladosporium* IgE antibody, U/mL | 11 | −0.78 (1.73) |
| *Dermatophagoides farinae* antigen IgE antibody, U/mL | 39 | −2.56 (6.53) |
| *D. pteronyssinus* antigen IgE antibody, U/mL | 37 | −1.47 (4.05) |
| Dog dander antigen IgE antibody, U/mL | 55 | −6.78 (12.14) |
| German cockroach antigen IgE antibody, U/mL | 7 | −1.02 (2.25) |
| IgE, *Aspergillus niger* allergen, U/mL | 2 | −0.64 (0.27) |
| IgE, Mouse urine protein allergen, U/mL | 15 | −2.84 (5.43) |
| *Penicillium notatum* IgE antibody, U/mL | 8 | −0.51 (0.91) |

*Definition of abbreviations:* BEC = blood eosinophil count; FEIA = fluorescence enzyme immunoassay; IgE = immunoglobulin E; SD = standard deviation.

Perennial aeroallergens listed in the table represent the allergens used in the FEIA test for serum-specific IgE to determine allergy status at baseline (positive allergy status defined as an FEIA test result of ≥0.35 kUA/L).

Baseline is the last nonmissing assessment on or before the first dose of tezepelumab at week 0.

# Table S5. Summary of SAEs Reported During the On-Treatment Period

| **SAE Category, *n* (%)** | **Tezepelumab 210 mg**  **Q4W**  **(*N* = 286)** |
| --- | --- |
| Any SAE | 28 (9.8) |
| Any adverse event resulting in death | 1 (0.3) |
| Any adverse event leading to treatment discontinuation | 6 (2.1) |
| SAE by system organ class and preferred term | |
| Infections and infestations | 13 (4.5) |
| Atypical pneumonia | 1 (0.3) |
| COVID-19 | 1 (0.3) |
| COVID-19 pneumonia | 1 (0.3) |
| Pneumonia | 4 (1.4) |
| Pneumonia (bacterial) | 3 (1.0) |
| Pneumonia (viral) | 1 (0.3) |
| Respiratory syncytial virus infection | 1 (0.3) |
| Sepsis | 1 (0.3) |
| Tooth infection | 1 (0.3) |
| Urosepsis | 1 (0.3) |
| Neoplasms (benign, malignant, and unspecified) | 3 (1.0) |
| Breast cancer | 1 (0.3) |
| Lung neoplasm (malignant) | 1 (0.3) |
| Squamous cell carcinoma | 1 (0.3) |
| Metabolism and nutrition disorders | 1 (0.3) |
| Lactic acidosis | 1 (0.3) |
| Nervous system disorders | 4 (1.4) |
| Cerebrovascular accident | 1 (0.3) |
| Paresthesia | 1 (0.3) |
| Seizure | 1 (0.3) |
| Transient ischemic attack | 1 (0.3) |
| Cardiac disorders | 1 (0.3) |
| Myocardial infarction | 1 (0.3) |
| Vascular disorders | 1 (0.3) |
| Arterial occlusive disease | 1 (0.3) |
| Respiratory, thoracic, and mediastinal disorders | 6 (2.1) |
| Acute respiratory failure | 1 (0.3) |
| Asthma | 5 (1.7) |
| Pneumothorax | 1 (0.3) |
| Pulmonary edema | 1 (0.3) |
| Gastrointestinal disorders | 2 (0.7) |
| Abdominal hernia | 1 (0.3) |
| Small intestinal obstruction | 1 (0.3) |
| Hepatobiliary disorders | 1 (0.3) |
| Cholecystitis acute | 1 (0.3) |
| Musculoskeletal and connective tissue disorders | 2 (0.7) |
| Arthritis | 1 (0.3) |
| Back pain | 1 (0.3) |
| Renal and urinary disorders | 1 (0.3) |
| Acute kidney injury | 1 (0.3) |
| General disorders and administration-site conditions | 2 (0.7) |
| Noncardiac chest pain | 2 (0.7) |

*Definition of abbreviations:* Q4W = every 4 weeks; SAE = serious adverse event.

The table includes SAEs with an onset date on or after the date of the first dose of tezepelumab until the final follow-up visit.

Participants with multiple occurrences in the same category are counted once per category regardless of the number of occurrences.

# Figure S1. PASSAGE study design.

Q4W = every 4 weeks; SC = subcutaneously.

# Figure S2. Patient disposition.

# Figure S3. Change from baseline to week 52 in pre- and post-BD FEV_1_ (*A*) and in FEV_1_ reversibility (*B*) in the overall population.

Values above the bars are the mean (SD) change from baseline to week 52. BD = bronchodilator; FEV_1_ = forced expiratory volume in 1 second; SD = standard deviation.

# Figure S4. Proportion of responders at week 52 for ACQ-6 (*A*), AIRQ (*B*), and SGRQ total (*C*) scores in the overall population, by asthma phenotype and in underrepresented populations.

Responders were defined as participants who achieved minimal clinically important difference thresholds for each outcome, i.e., an absolute decrease from baseline of ≥0.5 points for ACQ-6 score, ≥2 points for AIRQ score, ≥4 points for SGRQ total score. Values in parentheses are the number of responders among the number of participants with available data. ACQ-6 = Asthma Control Questionnaire-6; AIRQ = Asthma Impairment and Risk Questionnaire; BEC = blood eosinophil count; CI = confidence interval; COPD = chronic obstructive pulmonary disease; SGRQ = St George’s Respiratory Questionnaire; SUA = severe, uncontrolled asthma.

# Figure S5. Change from baseline to week 52 in SNOT-22 total score in participants with SUA with comorbid CRSsNP (*A*) and CRSwNP (*B*), and in TNSS among participants with SUA and a TNSS result indicating a severe symptom at baseline (*C*).

(*A*, *B*) SNOT-22 total score ranges from 0 to 110, higher scores indicate greater severity. The values above the bars are LS mean (95% CI) changes from baseline to week 52. SNOT-22 responders were defined as participants who achieved minimal clinically important difference threshold of ≥8.9 points. (*C*) TNSS ranges from 0 to 12, higher scores indicate greater severity. The values above the bars are mean (SD) changes from baseline to week 52. CI = confidence interval; LS = least-squares; SD = standard deviation; SNOT-22 = Sino-Nasal Outcome Test-22; TNSS = total nasal symptom score.

# Figure S6. Proportion of FPC responders at week 52 among participants with FPC at baseline (overall population, participants with SUA without comorbid mild-to-moderate COPD, participants with SUA with comorbid mild-to-moderate COPD) (*A*), and PGI-C response categories at week 52 among participants with SUA with comorbid mild-to-moderate COPD (*B*).

(*A*) Responders were defined as achieving the minimal clinically important difference threshold of an absolute decrease from baseline of ≥3 points. Values in parentheses are the number of responders among the number of participants with available data. (*B*) Of 57 participants with SUA with comorbid mild-to-moderate COPD at baseline, 43 had PGI-C data at week 52. COPD = chronic obstructive pulmonary disease; FPC = frequent productive cough; PGI-C = Patient Global Impression of Change; SUA = severe, uncontrolled asthma.

# Figure S7. CGI-C response categories (*A*) and mean TSQM-9 domain scores (*B*) at week 52 in the overall population.

TSQM-9 domain scores can range from 0 to 100, with higher scores representing higher satisfaction in that domain. CGI-C = Clinical Global Impression of Change; SD = standard deviation; TSQM-9 = Treatment Satisfaction Questionnaire for Medication-9.
